# An integrated analytical study of crayons from the original art materials collection of the MUNCH museum in Oslo

**DOI:** 10.1038/s41598-021-86031-6

**Published:** 2021-03-30

**Authors:** Jacopo La Nasa, Brenda Doherty, Francesca Rosi, Chiara Braccini, Frederique T. H. Broers, Ilaria Degano, Jordi Moles Matinero, Costanza Miliani, Francesca Modugno, Francesca Sabatini, Irina Crina Anca Sandu, Laura Cartechini

**Affiliations:** 1 Department of Chemistry and Industrial Chemistry, University of Pisa, Via G. Moruzzi 13, 56124 Pisa, Italy; 2 Istituto CNR di Scienze e Tecnologie Chimiche “G.Natta” CNR-SCITEC, Via Elce di Sotto 8, 01628 Perugia, Italy; 3 Istituto CNR per le Scienze del Patrimonio Culturale (CNR-ISPC), Via Cardinale Guglielmo Sanfelice 8, 80134 Napoli, Italy; 4 Department of Collection Care and Management, MUNCH, Edvard Munchs Plass 1, Sørenga, Postboks 3304, 0194 Oslo, Norway

**Keywords:** Chemistry, Materials science

## Abstract

Among the artists’ materials of the nineteenth century, pastel crayons merit scientific interest since their early commercial formulations are mostly unknown and, until now, have been considerably less studied with respect to other contemporary painting materials. In this framework, research herein reports the results of a comprehensive multi-analytical study of 44 pastel crayons of two recognized brands (LeFranc and Dr. F. Schoenfeld) from the Munch museum collection of original materials belonging to Edvard Munch. The integrated use of complementary spectroscopic and hyphenated mass-spectrometry techniques allowed the compositional profiles of the crayons to be traced providing the identification of the inorganic and organic pigments, the fillers/extenders and the binders. All crayons resulted to be oil- based and the binder was identified to be a mixture of a drying oil (safflower or linseed oil), palm oil or Japan wax and beeswax. Among others, pigments such as ultramarine, chrome yellows, Prussian blue, manganese violet, viridian and madder lake have been identified. A significant alignment in formulations of the brands was observed with the only exception of the greens which showed distinctive pigment and filler compositions. The analytical information provided for these commercial artists’ materials will be of great interest for academia, museum and other institutions hosting art collections dating from the same period and it will be used by the Munch museum to draw proper conservation strategies of its own artwork collections.

## Introduction

Edvard Munch (1863–1944), one of the fathers of modern expressionism in art history, was a polyvalent artist who experimented with a wide variety of materials and techniques throughout his artistic career.

Indeed, Munch’s art production took place during a renowned period of great innovation in chemical organic synthesis and industrial research. Vast numbers of new appealing painting materials became available between the end of the nineteenth century and the first decades of the twentieth century, and Munch explored their potentialities in his artworks, without neglecting the use of traditional materials.

An important part of the Munch Museum (MUNCH) collection in Oslo comprises the original artists’ materials^1^ used by Edvard Munch himself, of which around 300 items consist of pastel crayons of different brands. This part of the collection has never received the attention it would deserve as an important source of nineteenth century artist´s materials. For a complete characterization of the materials in this collection, for eventually dating them and understanding degradation mechanisms of paint formulations over time, in-depth analytical studies are needed. MUNCH is therefore developing a large core project aimed at providing new scientific knowledge of the museum collection of Munch’s paintings and original materials from his studio (including paint tubes and drawing materials), supported by a robust interdisciplinary collaboration among scientists, conservators, restorers and art-historians^1^.

The terms pastel, crayon or indeed pastel-crayon are seemingly interchangeable although technical and historical literature from the seventeenth centuries provides only the slightest distinction that pastel actually referred to the work of art or the medium and that crayon or pastel crayon indicated the actual tool^2^. This style of painting, used since the sixteenth century, enjoyed a resurrection during the industrial revolution due to the commercial availability of the portable ready to use crayons with palettes of bright and muted tones and hues. Crayons offered artists the possibility to spontaneously and conveniently paint with ready at hand dry colours in a stick form. Furthermore, they could be applied directly to a support where a work could quickly be taken up and even finished at pleasure. Without a doubt, crayons were incredibly appealing and useful for sketching compositions with precise recourse to fine colouring details which may fleetingly pass momentarily, and which may not be well remembered later. Whether soft, or semi-hard, and with rough chalk like or smooth oil or wax like surfaces, the pastel crayons were marketed to be used for sketching in or for providing precision details which would have been ideal for different kinds of supports. The early commercial formulations of pastel crayons still remain undisclosed^3,4^, and actually merit in depth analyses to elucidate the constituent materials which conferred them their specific physicochemical properties. In general, the chemical composition of pastel crayons has been considerably less studied than other artists’ materials, and very few published technical studies are available on the analysis of artworks containing them^3,5–8^. Along these lines and within the FUTURAHMA project (http://www.futurahma.it/en/home/) a study was undertaken to document and analyze artists’ materials and their rapid evolution at the beginning of twentieth century through archives and scientific analysis on Vittore Grubicy de Dragon’s documentary sources and artworks, and on Lefranc archives^9,10^. Previous studies on historical reproductions of paint materials^11^, and comparative studies of recipes and other information from manufacturing companies archives (e.g. the Winsor & Newton database^12^) highlight the relevance of the MUNCH’s collection of atelier materials for providing new insights and data on the history of paint materials in the nineteenth century^13^.

**Figure 1:**
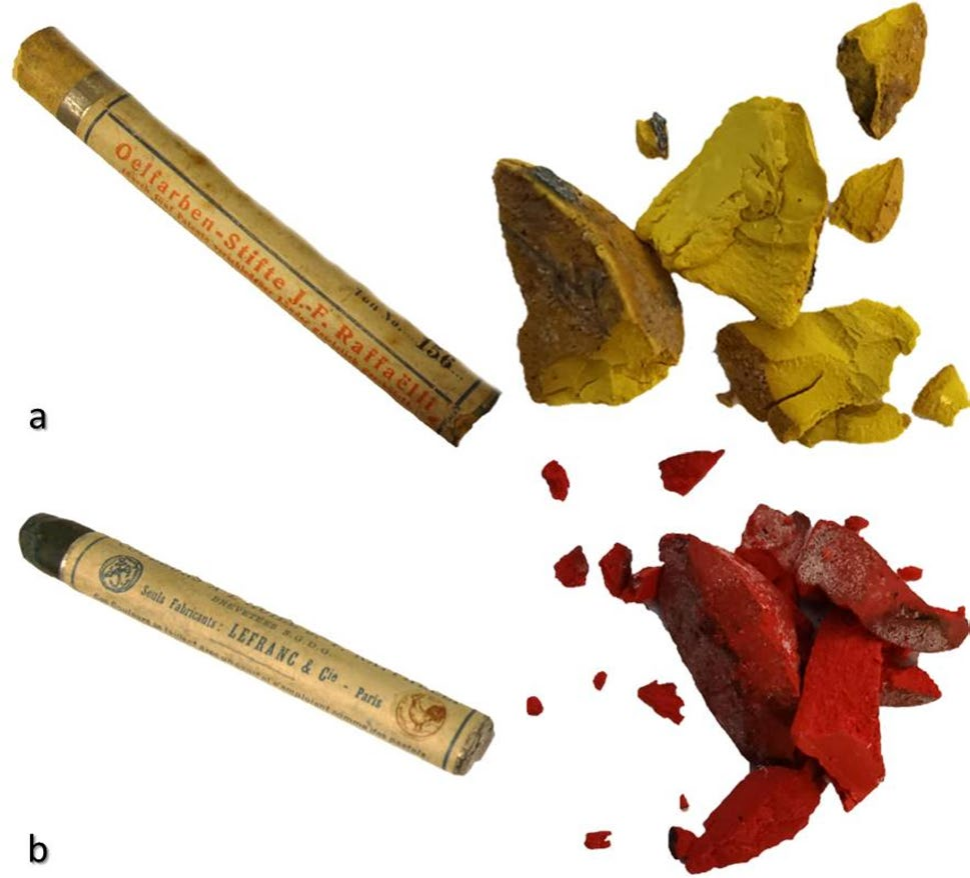
Two examples of pastels crayons and the micro-fragments collected for the analytical study: **(a)** yellow color from *Oljefarben-Stifte J. F. Raffaelli* of Dr. Fr. Schoenfeld; **(b)** red colour from *Couleurs a l'huile J.F. Raffaelli* of LeFranc.

The herein presented research project allowed a comprehensive multi-analytical study of several pastel crayons of two recognized brands (*Couleurs a l'huile J.F. Raffaelli* of LeFranc and *Oljefarben-Stifte J.F. Raffaelli* of Dr. F. Schoenfeld) from the Munch museum’s original materials collection (Fig. 1).

Specifically in this work, complementary analytical techniques, namely X-ray Fluorescence spectrometry (XRF)^14^, Fourier-transform InfraRed (FT-IR)^15^, Raman, and Surface Enhanced Raman (SERS) spectroscopies^16,17^, were applied for the identification of the inorganic and organic pigments, fillers and extenders. The characterization of the organic components was carried out at molecular level by analytical pyrolysis coupled with gas chromatography-mass spectrometry (Py-GC–MS)^18,19^, liquid chromatography coupled either with diode array detector and fluorescence detector (HPLC–DAD-FD) or high resolution mass spectrometry (HPLC-HRMS)^20,21^, and flow injection analysis-HRMS (FIA-HRMS)^22,23^ in order to achieve both the compositional profile of the binders and information on organic additives and pigments.

Since Munch mainly used commercial artist colors that were common and well-known among his contemporary artists, this study will be of a wider interest to academia, museum professionals and other institutions hosting art collections dating from the same period. Thus, the results of the analytical campaign will increase the knowledge of the formulation of pastel crayons produced by different manufacturers in the beginning of the twentieth century. This knowledge will have direct impact on the definition of proper conservation strategies of such materials in artworks.

## Materials and methods

### Samples

Forty-four pastel samples from two brands were analyzed from the MUNCH collection: 17 from the line *Couleurs a l'huile F. Raffaelli* by LeFranc manufacturer and 27 from the line *Oljefarben-Stifte J.F. Raffaelli* of the brand Dr. F. Schoenfeld, hereafter denoted as LF and DS, respectively. In Table 1 the samples are listed by color (red, brown, purple, blue, yellow, green, and grey/black), along with a brief summary of the results to evidence differences and similarities of the formulations (see “Results and discussion” herein and supplementary information online).

**Table 1:**
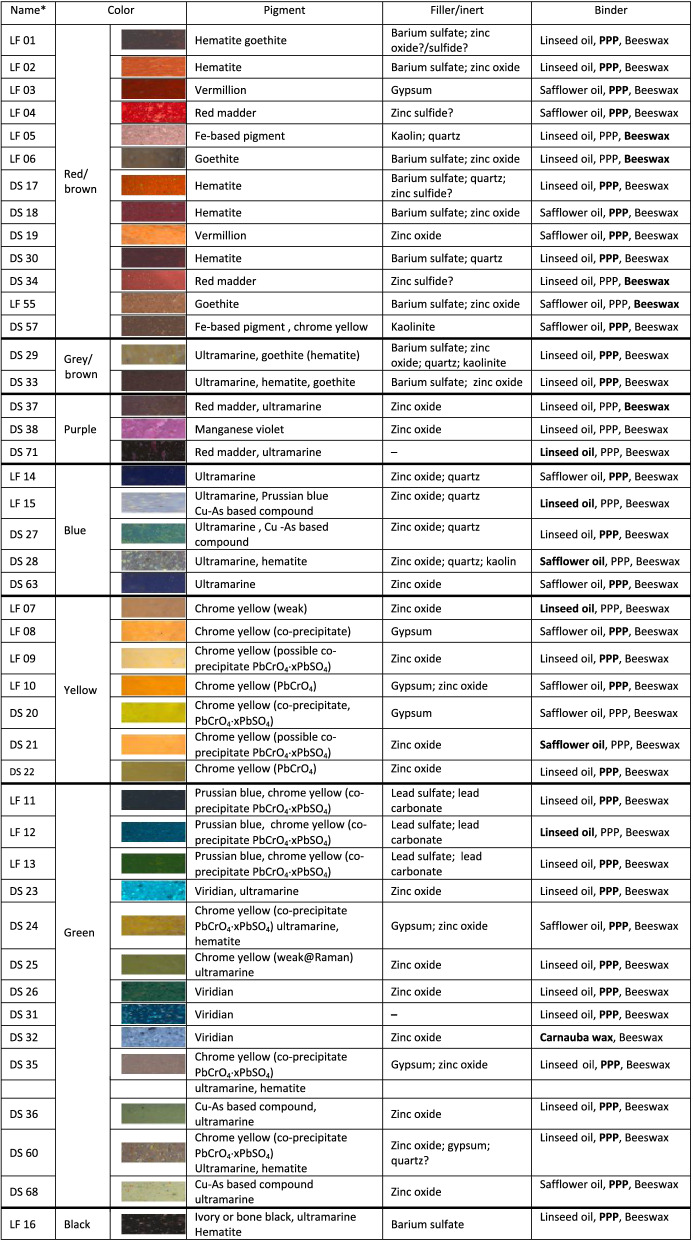
List of the 44 pastels and summary of the results from the integrated analytical approach. Colour images are from optical microscope photographs of the microsamples (64 × magnification).

Bold: most intese signals identified in the FIA-MS mass spectrum of the paint binder (PPP = tripalmitin).

*LF: Le Franc, *DS:* Dr.F. Schoenfeld.

### XRF

XRF measurements were performed directly on pastel samples by a portable XRF spectrometer from XGLab (Bruker) equipped with a rhodium anode tube operated at 100 μA and 40 kV. The X-ray beam was collimated on the samples by 1 mm pinhole collimator and acquisition time was 40 s. All the samples were analyzed by XRF.

### FT-IR

FT-IR spectra of the samples were recorded in transmission mode on micro-samples dispersed in pressed KBr pellets by a JASCO FT-IR 4100 spectrometer. The measurements were carried out in the 4000–400 cm^−1^ range at a resolution of 2 cm^−1^ using 100 scans. All the samples were analyzed by FT-IR.

### μ-Raman and SERS

μ-Raman spectra were collected using a laboratory JASCO NRS-3100 spectrophotometer with laser excitations at 514 nm (1200 l/mm grating) and/or 785 nm (800 l/mm grating). The confocal set-up permitted micro-samples to be observed with the objective 100 × where spectra could be recorded over the range 300–2000 cm^−1^ with an exposure time from 3 to 5 s and 7–10 accumulations. The laser power at the sample was maintained between 0.2 to 0.4 mW for λ514 nm and below 20 mW at λ785 nm. The instrument was calibrated using a polystyrene reference.

For the SERS measurements, a silver colloidal solution prepared via a modified Lee Meisel procedure on the reduction of silver nitrate (Aldrich silver nitrate 99.9%) by sodium citrate (Aldrich sodium citrate dehydrate 99%) was utilized^24^. As measured by UV–Vis (Hewlett Packard 8453 photodiode array spectrometer upon a 1:9 dilution with ultrapure water), the colloid observed a characteristic absorption maximum at 426 nm and FWHM of 110 nm. A 5 μL drop of magnesium sulphate aggregated colloid was placed directly onto the micro-samples, and/or onto the micro-samples following pre-treatment with hydrofluoric acid where indicated^25^. On addition of the colloid, spectra could be recorded and remained constant in quality until the evaporation of the liquid. Data was collected utilizing laser excitation at 514 nm, over the range 150–1800 cm^-1^ with exposure times from 2 to 10 s and 3–10 accumulations. Laser power was maintained between 0.6 and 2 mW with an overall spectral resolution of ~ 4 cm^-1^. All the samples were investigated by μ-Raman while SERS was applied for the analysis of the organic pigments in the samples LF04, DS71, DS37 and DS34.

### HPLC–DAD-FD and HPLC-ESI-Q-ToF

The HPLC system is constituted by a PU-2089 quaternary pump equipped with a degasser, an AS-950 autosampler, a MD-2010 spectrophotometric diode array detector (DAD) and a FP-2020 fluorescence detector (FD) equipped with a Xenon lamp (150 W), all Jasco International Co., Japan. The spectra acquisition parameters of the DAD were: 200–650 nm acquisition range, 0.8 s scan range, 4 nm resolution. The λex/λem selected for the fluorescence program used were: 350/550 nm for 12.0 min, 474/547 nm from 12.1 to 39.0 min. The gain selected was 1000 × . The injection volume was 20 μL.

The HPLC-ESI-Q-ToF system is composed an HPLC 1200 Infinity, a Jet Stream ESI-Q-ToF 6530 Infinity detector, and an Agilent Infinity autosampler (Agilent Technologies, Palo Alto, CA, USA). The injection volume was 4 μL and the io ionization mode was negative.

The mass spectrometer parameters and operative conditions, the analytical column and the chromatographic conditions selected for both the HPLC systems are reported in literature^26^.

The sample pre-treatment consisted in adding 300 μL of MeOH/HCl (30:1) solution to c.a. 2 mg of sample, extracting in ultrasonic bath at 60 °C for 60 min, filtrating with PTFE (0.45 μm) filters, evaporating under nitrogen flow, re-dissolving with 200 μL of dimethyl sulfoxide (DMSO, J.T. Baker, USA). In particular, samples LF04, DS71, DS37 and DS34 were analyzed (see supplementary information S1 online).

### Py-GC–MS

The analyses were performed using an EGA/PY-3030D (Frontier Lab, Japan) multi-shot pyrolyzer coupled with a 6890 N gas chromatography system with a split/splitless injection port and a 5973 mass spectrometer (Agilent Technologies, U.S.A.). The samples were placed directly in stainless steel cups on glass wool with 2 µL of HMDS before the analyses. The full Py-GC–MS conditions are reported in^27,28^ (see supplementary information S2 online). Pyrolysis was applied to 12 samples, namely LF03, LF04, LF07, LF9, LF12, LF15, DS21, DS23, DS28, DS34, DS37, DS71.

### FIA-ESI-Q-ToF

C.a. 0.1 mg of all the sample was subjected to extraction using a microwave oven Ethos One (Milestone, U.S.A.) (power 600 W), the full conditions adopted for the extraction are reported in^13,22^. Analyses of the lipid binders were carried out using a 1200 Infinity HPLC, coupled with a 6530 Infinity Q-ToF detector by a Jet Stream ESI interface (Agilent Technologies, U.S.A.). The FIA-MS conditions were the same as described in^22^.

The TAGs identification was performed according to dataset reported in^29,30^. Fatty acid abbreviations: B: behenyl (C_22:0_), A: arachidyl (C_20:0_), L: linoleyl (C_18:2_), O: oleyl (C_18:1_), S: stearyl (C_18:0_), P: palmityl (C_16:0_). For the oxidized acyl substituents: C_n_° of carbon atoms: n° of unsaturation, n° of OH.

Principal component analysis (PCA) was performed using as a dataset the ions at *m/z* 829.7 (PPP, [M + Na]^+^), 857.7 (PPS, [M + Na]^+^), 913.8 (SSS, [M + Na]^+^) for palm oil/japan wax, the ions at *m/z* 855.7 (POP, [M + Na]^+^), 881.7 (OOP, [M + Na]^+^), 883.7 (OSP, [M + Na]^+^), 911.7 (OSS, [M + Na]^+^) for the drying oils, and the ions at *m/z* 869.8, 897.8, 925.8, 941.8 (beeswax di-esters^22^) for beeswax. The ions were integrated and normalized, and the data analysis was performed on the covariance matrix using Xlstat 10.0 (Addinsoft, France) (see supplementary information S3 and table S1 online).

## Results and discussion

### Inorganic pigments and fillers

**Figure 2:**
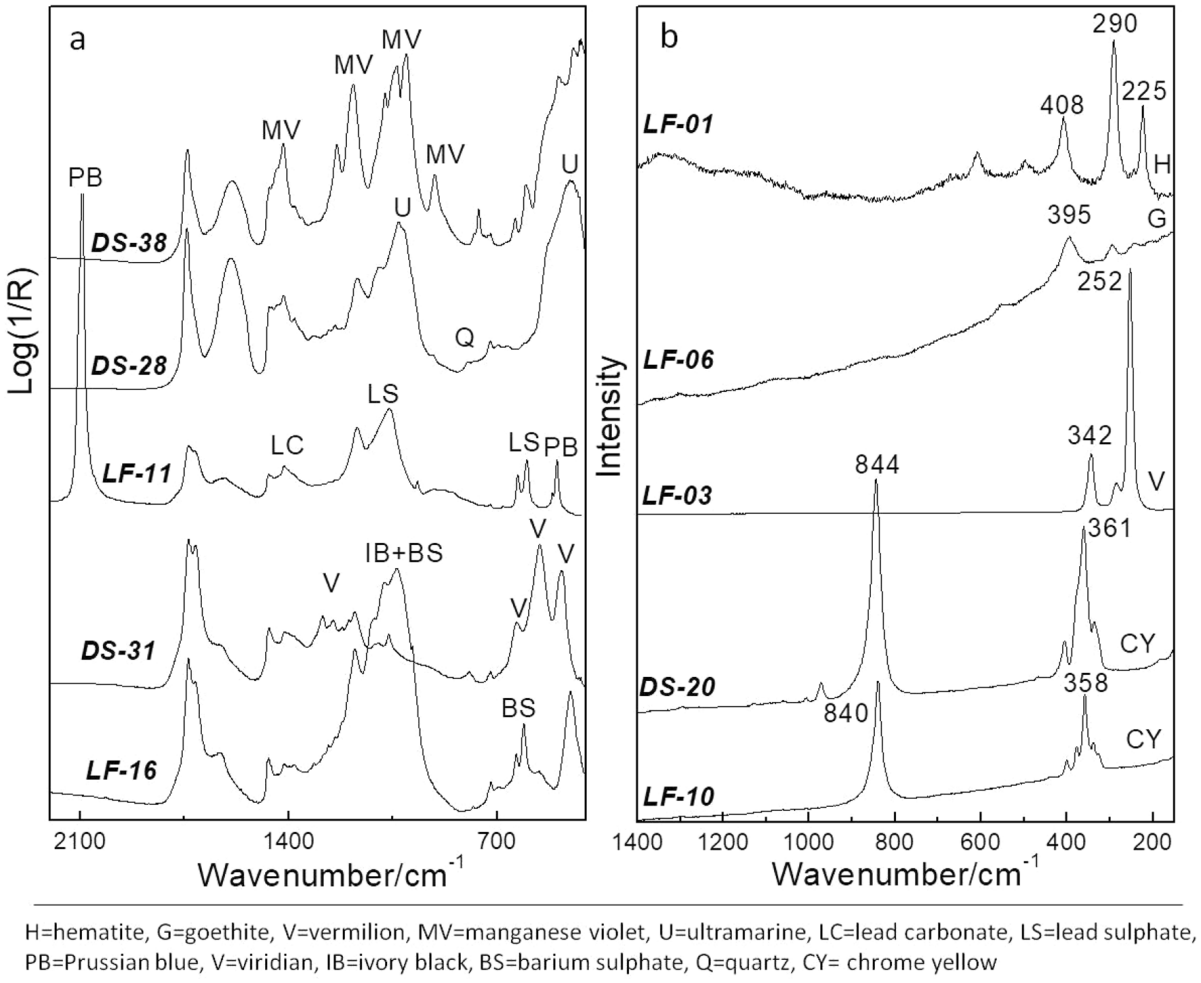
Examples of **(a)** transmission mode FT-IR spectra, and **(b)** μ-Raman (λ785 nm) spectra collected from selected LF and DS crayons.

Analyses by XRF, FT-IR and Raman spectroscopies were carried out on micro samples from all the 44 selected pastel crayons allowing for the characterization/identification of the inorganic materials (pigments, fillers and inerts—examples of spectral data shown in Fig. 2) and providing complementary information to the molecular analysis of the binder and of the organic pigments (see “Organic pigments” and “Binders”). A summary of the main results is reported in Table 1. Full XRF results are presented in supplementary table S2 online**.**

In general, fillers and inerts were not distinctive for the two brands: zinc oxide (ZnO) and barium sulphate (BaSO_4_, Fig. 2a, sample LF16) were the most common fillers (present also together); the use of zinc sulfide (ZnS) was indirectly inferred for the specific case of the red samples LF04 and DS34 where Zn and S were present as major elements in the XRF spectrum, while no distinctive spectral features of ZnO were observed by Raman or FT-IR. Similar hints were given by Raman and FT-IR for the red samples LF01 and DS17 where Zn and S were also detected by XRF, but in these cases the co-presence of BaSO_4_ hampered the univocal attribution of the sulphur XRF signal to ZnS, making its recognition uncertain. Gypsum (CaSO_4_⋅2H_2_O) was rarely found and used alone or in combination with ZnO. Kaolinite (Al_2_Si_2_O_5_(OH)_4_) and quartz (SiO_2_) were sporadically observed with Fe-based pigments; quartz was also found associated to ultramarine pigment (Na_8–10_Al_6_Si_6_O_24_S_n,_) in all the blue pastels from both brands. Notably, the deep and bright green hues of the LF brand showed the peculiar presence of lead sulphate and carbonate (Fig. 2a, sample LF11). In the samples, the presence of impurities accounts for minor XRF intensities of some elements, as for example Al, Si, K, Ca, Ti, Ba, Fe. Sr was generally found as vicarious ion of Ba.

Concerning the inorganic pigments, red and brown pastels from both brands were characterized by the presence of iron based pigments, namely iron oxides and hydroxides, mainly hematite, (αFe_2_O_3_), and goethite (αFeOOH)^31,32^] or vermillion (HgS), as shown by the Raman spectral profiles in Fig. 2b for samples LF01, LF06 and LF03, respectively.

In few samples, both from LF and DS, a red organic pigment was present as identified by SERS and HPLC–DAD-FD and HPLC-HRMS analyses (see “Organic pigments”). The same organic pigment was used to obtain the purple hue in two of the three analyzed purple pastels (one from LF and the others from DS) by admixture with ultramarine (Na_8–10_Al_6_Si_6_O_24_S_n,_). In the third purple sample from DS, manganese violet was clearly identified by XRF and FT-IR spectroscopy (Fig. 2a, sample DS38)^33^.

Ultramarine was ubiquitous in all the blue pastels from LF and DS (Fig. 2a, sample DS28^34,35^), with both brands featuring quartz as accessory phase. Ultramarine was used alone or in mixture with other pigments such as Prussian blue (formally Fe^III^_4_[Fe^II^(CN)_6_]_3_·*x*H_2_O^36^) in a pastel from LF (LF15), or a Cu-As based pigment (not identified, see below), in either LF or DS samples. Ultramarine was also detected in the grey/black and green pastels.

Chrome yellow is the main yellow pigment for both the brands, highlighted by Raman spectroscopy either as lead chromate (PbCrO_4_, Fig. 2b, sample LF10) or as co-precipitate of lead chromate and sulfate (PbCrO_4_⋅xPbSO_4_ with x < 0.5, Fig. 2b, sample DS20^37^). The identification of the co-precipitate form is important to establish the lightfastness of the pigment, since for chrome yellow co-precipitates richer in sulfates with x > 0.5 the orthorhombic structure is favored, which shows high susceptibility toward darkening^38^.

Concerning the green pastels, the pigments used to obtain the green hues provided the most interesting results for discriminating the two brands, and confirmed the use of different formulations as already evidenced by the peculiar presence of lead sulphate and carbonate as fillers in the LF samples. All the LF green samples (LF11, LF12, and LF13) contained Prussian blue in combination with chrome yellow (of the co-precipitate type: PbCrO_4_⋅xPbSO_4_, with x < 0.5) but it is noted that in these samples the IR bands of lead sulphate are very intense which suggests another origin for this compound beyond the co-precipitate pigment^39^. Instead, in the case of the DS green samples, different pigments were used: (i) viridian (Cr_2_O_3_·(xH_2_O), Fig. 2b sample DS31^40^), alone or combined with ultramarine; (ii) ultramarine admixed with co-precipitate PbCrO_4_⋅xPbSO_4_ (Raman signals too weak for determination of x) and sometimes with hematite, to modulate the green hue; (iii) a Cu-As based pigment in mixture with ultramarine. The Cu-As pigment was detected by XRF in the samples LF15, DS27, DS36, DS68 but unfortunately FT-IR and Raman spectroscopies were unable to provide its identification neither as Scheele’s green (mixture of copper(II)-arsenites) nor as Emerald green (copper(II)-acetoarsenite). This can be explained with the small pigment amount and the spectral overlapping with respect to the filler ZnO and the binder which hampered spectral recognition^41^.

The only black sample here analyzed is from the LF brand where ivory/bone black was clearly found by FT-IR spectroscopy (Fig. 2a, sample LF16^42^).

### Organic pigments

**Figure 3:**
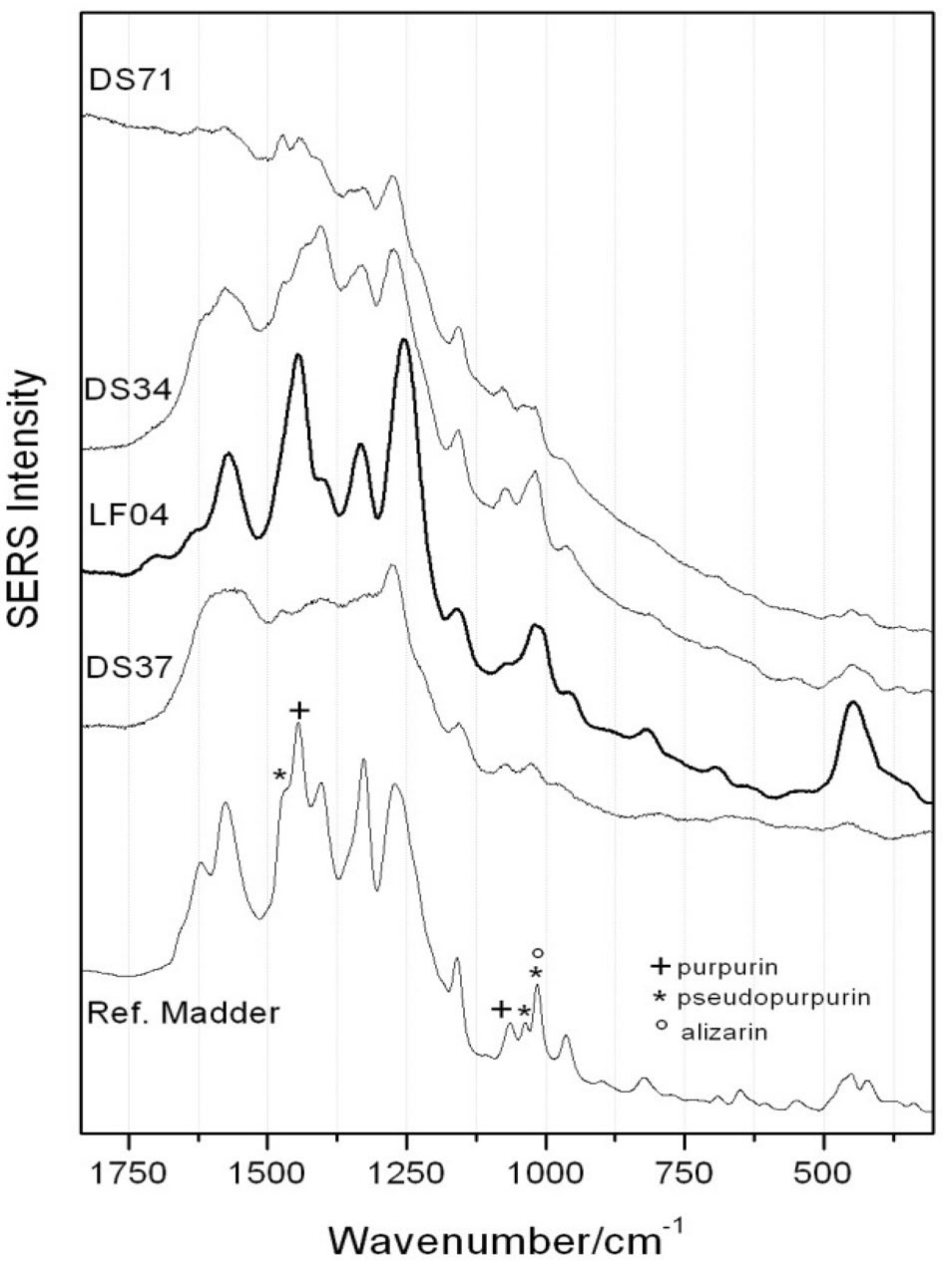
SERS spectra of pastels DS71, DS34, LF04, DS37 together with reference Madder lake highlighting marker bands of main madder constituents: alizarin (°), purpurin ( +) and pseudopurpurin (*).

The nature of the red organic pigments in pastels LF04, DS71, DS37 and DS34 was qualitatively investigated by colloidal SERS where results could only be obtained after hydrolysis of the micro-samples. This suggests that the organic colorant was efficiently complexed to its inorganic substrate, possibly alum-based in accordance with the characteristic broad FTIR spectra, with features in the low wavenumber range 400–750 cm^-1^ (data not reported)^43^, thus reflecting a skilled pigment production by both brands. The overall quality of the spectra does however differ (Fig. 3): LF04 and DS34 seemingly interact better with the colloidal solution whereas the enhancement for DS71 and DS37 is likely hampered by the further presence of ultramarine, added to obtain the purple hue, as aforementioned in “Inorganic pigments and fillers”. The source of the organic color could be assigned to natural vegetal anthraquinones. In Fig. 3, it is possible to appreciate the spectral profiles of samples DS71, DS34, LF04, DS37, shown with a ref. madder for comparison. Evidenced marker bands and overall features pertaining to madder constituents (alizarin, purpurin and pseudopurpurin) suggest a formulation rich in purpurin and pseudopurpurin^9,44^.

It is known that madder, extracted since antiquity from the roots of *Rubia tinctorum* and other species^45^, continued to be used in early synthetic pigment production^7,46,47^.

**Figure 4:**
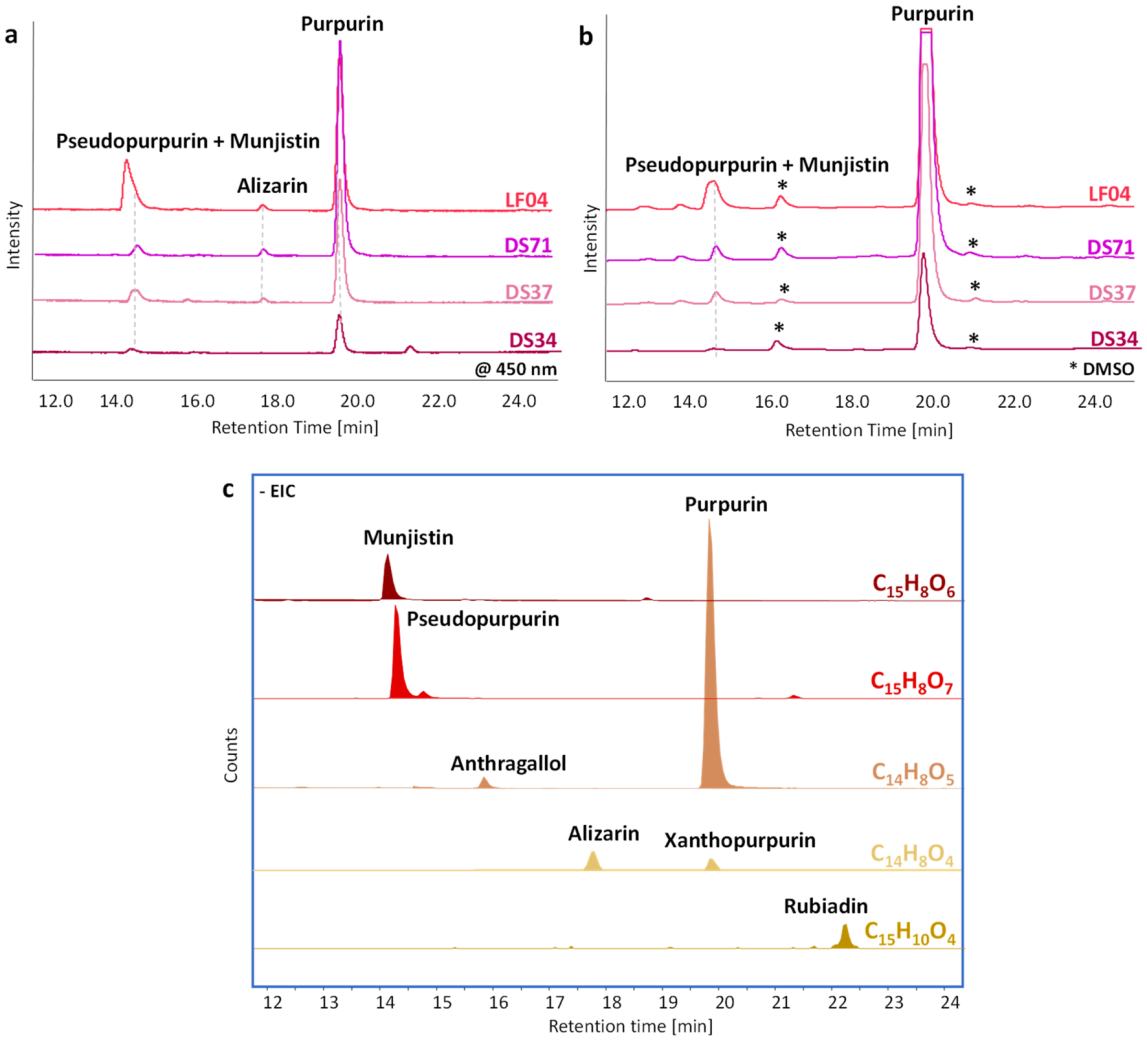
**(a)** HPLC–DAD acquired at 450 nm of LF04, DS71, DS37 and DS34 extracts; **(b)** HPLC-FD of LF04, DS71, DS37 and DS34 extracts; **(c)** HPLC-ESI-Q-ToF extract ion chromatograms (EICs) of anthraquinones (ions corresponding to the [M–H]^−^ ions for the molecular formulas C_15_H_8_O_6_, C_15_H_8_O_7_, C_14_H_8_O_5_, C_14_H_8_O_4_, C_15_H_10_O_4_) from the extracts of sample LF04. Negative acquisition mode.

The HPLC–DAD-FD chromatographic profiles of red pastels LF04, DS71, DS37 and DS34 (Fig. 4a,b) consistently highlight the presence of purpurin, munjistin and pseudopurpurin as main compounds and anthragallol and alizarin as minor ones. The identification of these compounds was unequivocally confirmed by high resolution mass spectrometry and other minor anthraquinoid compounds, namely xanthopurpurin and rubiadin, were also detected. All the HPLC-ESI-Q-ToF Extract Ion Chromatograms (EIC) showed a very similar composition (sample LF04 is shown in Fig. 4c), even if slight differences can be observed in the semi-quantitative profile. LF04 and DS037 show a very similar profile, with 70% purpurin and 17% munjistin, evaluated on the Extract Ion Chromatograms (EICs); DS071 has a higher content of purpurin (80%) and lacks pseudopurpurin. The most different sample is DS034, which contains the lower amount and number of components, peaking at purpurin (90%).

Despite the differences, the presence of several anthraquinoids typical of vegetal source (pseudopurpurin, munjistin and rubiadin) and the absence of anthraquinoid species usually formed as by-products during alizarin synthesis^47^ suggested that all the pastels contain indeed natural lake pigment extracted from madder root^48^, ruling out the use of synthetic alizarin and synthetic purpurin. The high abundance of purpurin and pseudopurpurin with respect to alizarin suggests that the lake was obtained from purified madder roots, according to Kopp’s purpurin process^49^.

The detection of madder-based lakes is consistent with the results achieved in our previous investigations performed on LeFranc archive materials, where Kopp’s purpurin was identified in artists’ materials dated 1890^47^.

### Binders

FT-IR spectroscopy was useful to provide for a general survey of the main binder components present in the examined crayons, preliminary to the in depth characterization at molecular level carried out by chromatographic and mass spectrometric techniques.

The FT-IR analyses evidenced a wax component in all the 44 samples along with variable amounts of a lipid component^50,51^. This was evidenced in the FT-IR spectra by a broadening of the CH stretching bands (3000–2700 cm^-1^) which occurs in lipids with respect to waxes and paraffins due to a greater disorder of the aliphatic hydrocarbon chains. FT-IR measurements were also useful to highlight the presence of pigment/binder interactions in all the samples containing ZnO. In these samples, in fact, a broad spectral band at about 1590 cm^-1^, assigned in literature to the formation of a zinc ionomer in ZnO oil paint film^52^, was observed (Fig. 2a, sample DS28 and DS38); this is to be explained with the presence in the crayons of a drying oil component that during the autoxidation process reacted with Zn ions from the pigment through the newly formed acid groups^53^. In few cases the presence of zinc soaps (possibly Zn stearate/palmitate^54^) was also evidenced by FT-IR (samples LF06, DS18, DS33, LF14, DS63, DS68).

FT-IR results were supplemented by Py(HMDS)-GC–MS with in situ derivatization with HMDS analyses performed on twelve samples (30% of the set). The analysis allowed fatty acids to be detected in all samples, while excluding the presence of proteins and polysaccharides. This demonstrates that the crayons under study are «oil pastels» or «wax pastels», as opposed to those crayons based on a polysaccharidic media, quite common in the same period^37^. The chromatographic profiles clearly indicate the presence of beeswax in all the analyzed samples, pointed out by the peak of the molecular marker 15-hydroxy-hexadecanoic acid^55^, admixed with at least another lipid binder. The mixed lipid materials occur in different relative amounts in the samples, as suggested by FT-IR and indicated by the variability in the observed fatty acid profiles. In all the samples palmitic acid (hexadecanoic) is the most abundant fatty acid, followed by stearic acid (octadecanoic). Beeswax pyrolysis profile is particularly rich in palmitic acid, being this wax mainly composed by palmitic acid monoesters (c.a. 50%)^56^. Japan wax^57^ and palm oil^13^, previously characterized in different works of art by Edvard Munch, also feature pyrolysis profiles rich in palmitic acid, being both mainly composed by palmitoyl containing glycerides. Moreover, the pyrograms feature variable amounts of dicarboxylic acids with 8, 9, and 10 carbon atoms (suberic, or α,ω-octanedioic; azelaic, or α,ω-nonanedioic; sebacic, or α,ω-decanedioic). This is a recognized chemical signature of aged drying oils, being such dicarboxylic acids formed during the curing and the ageing of highly unsaturated triglycerides bearing insaturations in the position 9 of the acyl chains^55^. Thus, the presence of these diacids reflects the degree of oxidation of the drying oil that can be connected with the sample composition as well as to the heterogeneous degree of oxidation in the portion of the same crayon stick (e.g., surface vs bulk).

**Figure 5:**
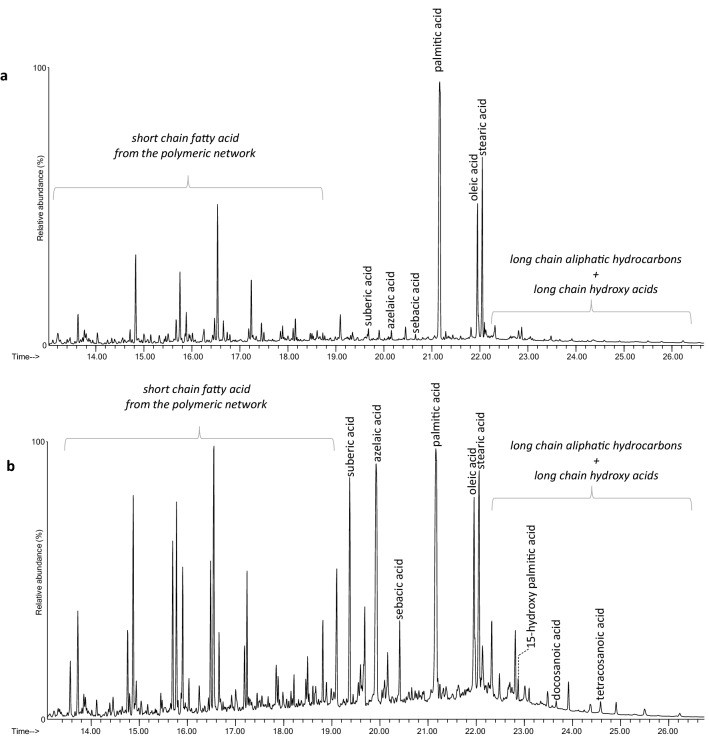
Py(HMDS)-GC–MS chromatograms obtained for a sample collected from crayon DS37 **(a)** and one from crayon DS34 **(b)**.

Figure 5 shows the Py-GC–MS chromatograms from one of the samples, DS34, which is particularly rich in dicarboxylic acids, and DS37, which was characterized by traces of these species. Finally, the pyrogram of only one of the samples—sample DS32—featured traces of methyl phenol and cinnamic acid derivatives, non-specific species that could derive from the addition of a phenolic or polyphenolic plant or animal (e.g., propolis) derivative in the formulation of this pastel. The hypothesis of their origin from Carnauba (known to contain cinnamic acid derivatives)^58^ was excluded on the basis of the FIA-ESI-Q-ToF results reported below.

The whole set of 44 samples were analyzed by FIA-ESI-Q-ToF after extraction aided by microwaves, but without any hydrolysis pretreatment. In the adopted conditions, the obtained mass spectra provide information on the profile of extractable monoacylglycerols (MAGs), diacylglycerols (DAGs) and triacylglycerols (TAGs) and of high molecular weight wax esters^22^. The determination of acylglycerols yields information on the botanical origin of oils and lipids, exploiting the higher specificity of the TAG profile and of high molecular weight wax esters compared to the less specific fatty acid profile obtained by GC–MS after saponification and Py(HMDS)-GC–MS^42^.

All samples contain: high molecular weight esters typical of beeswax^22^; unsaturated PPP triglyceride; oxidized TAGS in different relative amounts. In particular, all the spectra show the molecular ions attributed to the triglycerides of palmitic and stearic acid (see Sect. 2.6 for the abbreviations) : PPP (*m/z* 829.7, [M + Na]^+^), PPS (*m/z* 857.7, [M + Na]^+^), PSS (885.7, *m/z* [M + Na]^+^), and SSS (913.8, *m/z* [M + Na]^+^), consistently with the high amounts of palmitic and stearic acids observed in the Py-GC–MS chromatograms after thermal degradation of ester bonds and trimethylsilylation. These TAGs are possible markers of either Japan wax or oxidized palm oil.

**Figure 6:**
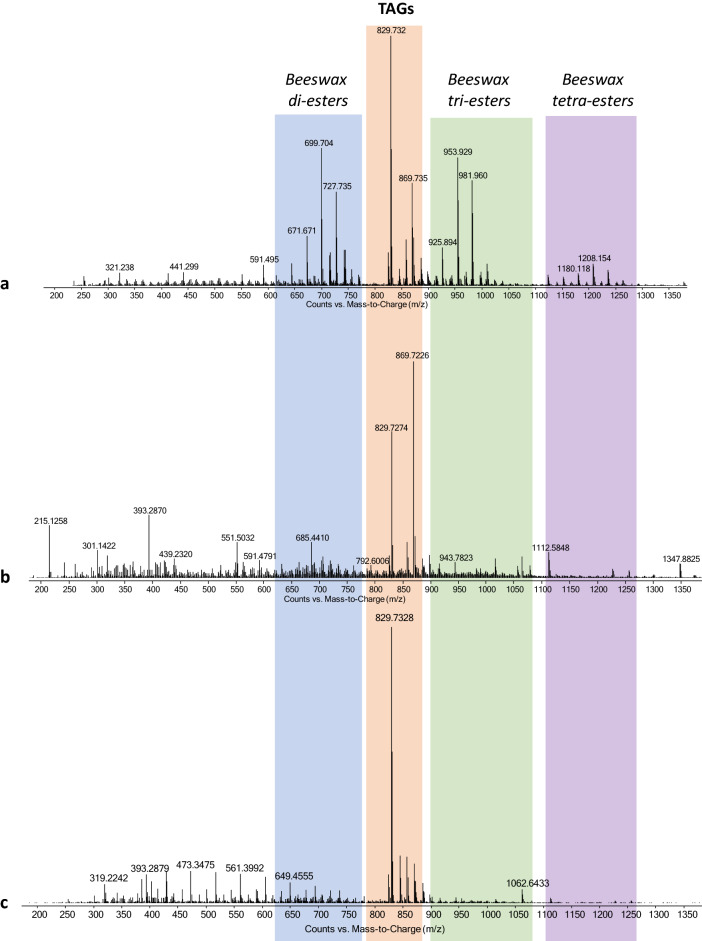
FIA-ESI-Q-ToF mass spectra of the extracts of samples DS37 **(a)**, LF07 **(b)**, and DS019 **(c)**.

The oxidized TAGs containing residual double bonds are ageing products of the easily oxidizable linoleyl-containing TAGs, abundant in highly polyunsaturated drying oils as linseed oil, whose presence was already hypothesized based on the amount of dicarboxylic acids in the Py-GC–MS chromatograms. In detail, FIA-ESI-Q-ToF mass spectra show the presence of species C_18:2,OH_PP (*m/z* 869.7, [M + Na]^+^), C_18:2,OH_OP (*m/z* 895.7, [M + Na]^+^), C_18:2,OH_C_18:2,OH_P (*m/z* 909.7, [M + Na]^+^), and C_18:2,OH_C_18:2,OH_S (*m/z* 937.7, ([M + Na]^+^). Among the unoxidized TAG portion of the pastels, the further presence of POP (*m/z* 855.7, [M + Na]^+^) and OSP (*m/z* 883.7, [M + Na]^+^) allowed us to positively identify the presence of linseed oil in 27 pastels, while the identification of traces of ALO (*m/z* 935.8, [M + Na]^+^), AOO (*m/z* 937.8, [M + Na]^+^), and BOO (*m/z* 965.8, [M + Na]^+^) in 15 pastels highlights the presence of safflower oil (Table 1)^29^. No correlation is observed between the trademark and the botanical origin of the oil used in the pastel crayons’ formulations. The mass spectra obtained for samples DS37, LF07, and DS019 are provided in Fig. 6 as example, showing the *m/z* fragments related to the high molecular weight esters typical of beeswax, and of TAGs.

**Figure 7:**
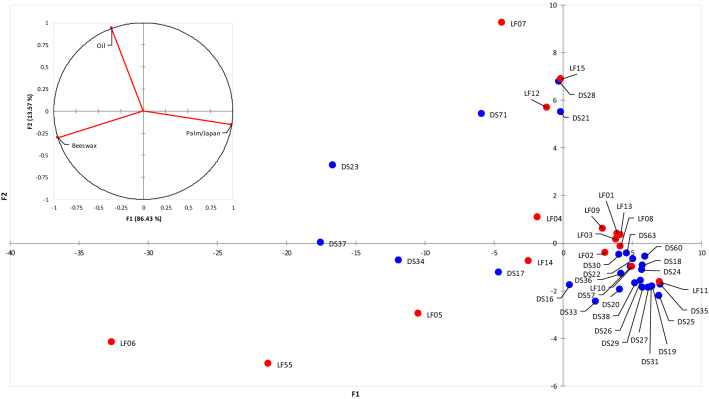
PCA score and loading plots obtained by processing the FIA-ESI-Q-ToF raw data (DS032 not included). Red: LeFranc; blue: Dr. F. Schoenfeld.

In conclusion, the profiles highlight the presence of three lipid materials: beeswax and palm oil/Japan wax (solid binders) and a drying oil (liquid additive that becomes solid with curing and oxidation). The relative amount of the three lipids is quite variable in the set of the investigated samples. Moreover, the relative amount of the three components cannot be ascertained by FIA-ESI-Q-ToF, since different compounds have different ionization rates. Thus, to evaluate the similarities and differences among the composition of the whole set in terms of their lipid content, multivariate data analysis based on principal components (PCA) was used (Fig. 7). Specific ions (See “FIA-ESI-Q-ToF” for more details) were selected to evaluate the relative amounts of beeswax, a siccative oil, and tripalmitin (which can derive either from palm oil or Japan wax).

Six samples (LF06, LF55, LF05, LF14, DS34 and DS17) with negative values of PC1 and PC2 contain relatively higher signals ascribed to beeswax, while eight samples at negative values of PC1 and positive values of PC2 contain a relatively higher signals typical of a drying oil (LF04, LF12, LF07, LF15, DS23, DS71, DS28 and DS21).

Nevertheless, no classification in function of the pigment/binder composition or the producer could be performed, even if most of the DS samples are characterized by more negative values of PC2 in respect to the LeFranc pastels. For instance, LF15 and DS28 feature the same pigment and same composition of the binder even if they are from two different producers. Sample DS32 was characterized by a FIA-MS spectrum and thus a composition that is completely different from any of the others, as already highlighted by the pyrogram. The occurrence of methyl phenol and cinnamic acid cannot be ascribed to carnauba wax^51^, since the FIA-MS profile of DS32 does not match with those of reference carnauba, nor with any of the available reference materials.

## Conclusions

Forty-four crayons belonging to the MUNCH collection, from 2 brands (17 from *Couleurs a l'huile J.F. Raffaelli* produced by LeFranc and 27 from *Oljefarben-Stifte J.F. Raffaelli* produced by Dr. F. Schoenfeld) covering different colors were analyzed through a multi analytical approach for characterizing their inorganic and organic components.

These crayons can be assigned to the category of oil crayons, as the main binding media identified are beeswax and one or more sources of triglycerides. The binders included beeswax, palm oil or Japan wax, and a drying oil constituent, specifically linseed oil and/or safflower oil. PCA analysis of FIA-ESI-Q-ToF data was used to compare the samples semi-quantitatively, highlighting the different relative abundance of the components in the crayon set.

Several synthetic pigments were identified such as ultramarine, chrome yellow, Prussian blue, manganese violet and viridian; the madder lake pigment found in some LF and DS red and purple crayons is of vegetal origin. The analyses identified zinc oxide and barium sulphate as the most common fillers (present also together) for both brands, while gypsum was less commonly found.

The overall results showed no significant differences between the two brands for what concerns the formulation of the organic binder and the pigments; the only exception are the greens. In fact, the deep and bright green hues of the LF brand showed the distinctive presence of a mixture of Prussian blue with chrome yellow added with lead sulphate and carbonate. Differently, the green hues of the DS brand consisted of different pigment formulations containing viridian, admixtures of ultramarine with lead chromate or Cu-As based pigments.

Lastly, the identification of different chrome yellow pigments of the type lead chromate or the co-precipitate of lead chromate and sulfate provided further clues for the conservation of these materials in relation to the different lightfastness of the pigment as a function of the lead sulfate content.

Overall, the obtained results represent an important contribution to the knowledge of pastel formulations in the nineteenth century. Thus, the Munch Museum and other similar collections will benefit from these advances in the processes of cataloguing and preservation of original artists’ materials and Munch artworks.

## Supplementary Information

Supplementary Information.

## Data Availability

The datasets generated during and/or analysed during the current study and not included in this paper are available from the corresponding author on reasonable request.
