# Supplementary material for: An integrated analytical study of crayons from the original art materials collection of the MUNCH museum in Oslo
Source: Sci Rep. 2021 Mar 30;11:7152. doi: 10.1038/s41598-021-86031-6 (PMC8010065; doi:10.1038/s41598-021-86031-6)
Supplement: Supplementary file 1 — Supplementary Information. [file 41598_2021_86031_MOESM1_ESM.docx]

**Supplementary Information**

**An integrated analytical study of crayons from the original art materials collection of the MUNCH museum in Oslo**

Jacopo La Nasa, Brenda Doherty^*^, Francesca Rosi^*^, Chiara Braccini, Frederique T.H. Broers, Ilaria Degano, Jordi Moles Matinero, Costanza Miliani, Francesca Modugno, Francesca Sabatini, Irina Crina Anca Sandu, Laura Cartechini

*****corresponding authors e-mails**:** [brenda.doherty@cnr.it](mailto:brenda.doherty@cnr.it); [francesca.rosi@cnr.it](mailto:francesca.rosi@cnr.it)

The following details supplement section 2: Materials and Methods

**S.1**

**HPLC-DAD-FD and HPLC-ESI-Q-ToF**

The HPLC system consists of a PU-2089 quaternary pump equipped with a degasser, an AS-950 autosampler, an MD-2010 spectrophotometric diode array detector (DAD) and an FP-2020 fluorescence detector (FD) equipped with a Xenon lamp (150 W), all Jasco International Co., Japan. ChromNav software was used to carry out data acquisition and data analysis. The DAD detector operated with spectra acquisition in the range of 200–650 nm every 0.8 s with 4 nm resolution. The λex/λem selected for the fluorescence program used were: 350/550 nm for 12.0 min, 474/547 nm from 12.1 to 39.0 min. The gain selected was 1000×.

For HPLC-ESI-Q-ToF analyses, an HPLC 1200 Infinity was used, coupled to a Jet Stream ESI-Q-ToF 6530 Infinity detector, and equipped with an Agilent Infinity autosampler (Agilent Technologies, Palo Alto, CA, USA). MassHunter® Workstation Software (B.04.00) was used to carry out mass spectrometer control, data acquisition, and data analysis. The mass spectrometer operated in ESI negative ionization mode and the working conditions were: drying gas N_2_ (purity>98%) temperature 350 °C and 10 L/min flow; nebulizer gas pressure 35 psi; capillary voltage 4.5 KV; sheath gas temperature 375 °C and 11 L/min flow; fragment or voltage175 V; nozzle voltage 1000 V; skimmer voltage 1000 V; octapole RF voltage 750 V. High resolution MS and MS/MS spectra were acquired in negative mode in the range 100-1000 m/z at a scan rate of 1.04 spectra/s (CID voltage 30 V, collision gas N_2_, purity 99.999%). The FWHM (Full Width Half Maximum) of quadrupole mass bandpass used during MS/MS precursor isolation was 4 m/z. Auto-calibration was performed daily using Agilent tuning mix HP0321 (Agilent Technologies) prepared in acetonitrile.

The chromatographic separation was performed in both systems on an analytical reversed-phase column Poroshell 120 EC-C18 (3.0×75 mm, particle size 2.7 μm,) with a pre-column Zorbax (4.6×12.5 mm, particle size 5 μm) both from Agilent Technologies (Palo Alto, CA, USA). The eluents were A: formic acid (FA; 98% purity, J.T. Baker, USA) (0.1% v/v) in LC-MS grade water and B: formic acid (0.1% v/v) in LC-MS grade acetonitrile. The flow rate was 0.4 mL/min and the program was: 15% B for 2.6 min, then to 50% B in 13.0 min, to 70% B in 5.2 min, to 100% B in 0.5 min and then hold for 6.7 min; re-equilibration took 11 min. During the separation, in both systems, the column was thermostated at 30 °C. The injection volume was 20 μL and 4 μL for the HPLC-DAD-FD and LCMS/MS systems, respectively.

The sample pre-treatment consisted in adding 300 μL of MeOH/HCl (30:1) solution to c.a. 2 mg of sample, extracting in ultrasonic bath at 60 °C for 60 minutes, filtrating with PTFE (0.45 μm) filters, evaporating under nitrogen flow, re-dissolving with 200 μL of dimethyl sulfoxide (DMSO, J.T. Baker, USA).

**S.2**

**Py-GC-MS**

Solid samples were analyzed by pyrolysis interfaced with gas-chromatography with mass spectrometric detection. Analyses were performed using a multi-shot pyrolyzer EGA/PY-3030D (Frontier Lab, Japan) coupled with a 6890N gas chromatography system with a split/splitless injection port and combined with a 5973 mass selective single quadrupole mass spectrometer (Agilent Technologies, U.S.A.). The samples were placed in deactivated stainless-steel sample cups on glass wool. The samples were added with HMDS and placed on top of the pyrolyzer at room temperature, and then quickly introduced in the pyrolysis chamber. The pyrolysis conditions were optimized as follows: pyrolysis chamber temperature 550°C, interface 280 °C. The GC injector temperature was 280°C. The GC injection was operating in split mode and the best analytical results were obtained with a split ratio of 1:10. The chromatographic separation of pyrolysis products was performed on a fused silica capillary column HP-5MS (5% diphenyl-95% dimethyl-polysiloxane, 30 m x 0.25 mm i.d., 0.25 μm film thickness, J&W Scientific, Agilent Technologies), preceded by 2 m of deactivated fused silica pre-column with internal diameter of 0.32 mm. The chromatographic conditions for the analysis were: 36°C for 10 min, 10°C min^-1^ to 280°C, 300°C for 2 min, 15°C min^-1^ to 300°C. The carrier gas was helium (purity 99.9995%) with a gas flow set in constant flow mode at 1.2 mL min^-1^. The MS parameters were as follows: electron impact ionization (EI, 70 eV) in positive mode; ion source temperature 230°C; scan range 50-700 m/z; interface temperature 280°C. Perfluorotributylamine (PFTBA) was used for mass spectrometer tuning. MSD ChemStation (Agilent Technologies) software was used for data analysis and peak assignment was based on a comparison with with literature mass spectra, standard compounds previously analysed in the same conditions, and libraries of mass spectra (NIST 20).

**S.3**

**FIA-ESI-Q-ToF**

The reference raw materials and archaeological organic residues were submitted to extraction using a Milestone microwave Ethos One system with 300µL of a chloroform:hexane (3:2) solution for 25 min at 80°C with an irradiation power of 600 W. The extracts were dried, diluted in the elution mixture, and filtered on 0.45 µm PTFE filters (Grace Davison Discovery Sciences, USA) before the injection. The amounts of samples subjected to the extraction procedure were in the range 50-150 µg.

The analysis was carried out using a 1200 Infinity HPLC coupled to a Jet Stream ESI interface with a Quadrupole-Time-of-Flight tandem mass spectrometer 6530 Infinity Q-ToF (Agilent Technologies, US).

The eluents were methanol:water (85:15) and *iso*-propanol; the flow rate was 0.2 mL/min and the injection volume was 1 µL.

The ESI operating conditions were: drying gas (N_2_, purity >98%): 350 °C and 10 L/min; capillary voltage 4.5 KV; nebulizer gas 35 psig; sheath gas (N_2_, purity >98%): 375 °C and 11 L/min. High resolution MS and MS/MS spectra were acquired in positive mode in the range 100-3200 m/z.

For the MS^2^ experiments the fragmentor was kept at 200 V, nozzle voltage 1000 V, skimmer 65 V, octapole RF 750 V. The voltage for the CID experiments was optimized for the different mass ranges in the range 20-100 V [1-4].

The collision gas was nitrogen (purity 99.999%). The data were collected by target MS/MS acquisition with a MS scan rate of 1.03 spectra/sec and MS/MS scan rate of 1.05 spectra/sec; only four precursors were acquired per analysis. The acquisition time for each MS/MS experiment was 1.5 min.

The mass axis was calibrated daily using the Agilent tuning mix HP0321 (Agilent Technologies, US) prepared in acetonitrile. The TAGs identification was performed according to dataset reported in literature [3, 5] (Table S.1).

Table S1 - Acylglycerols identified by the HPLC-ESI-O-ToF analysis with formula, parent and product ions, and proposed fragmentation in their tandem mass spectra. The identified acyl species are named according to the following fatty acid abbreviations: B: behenyl (C_22:0_), A: arachidyl (C_20:0_), L: linoleyl (C_18:2_), O: oleyl (C_18:1_), S: stearyl (C_18:0_), P: palmityl (C_16:0_). For the oxidized acyl substituents: C_n°of carbon atoms:n° of unsaturation, n° of OH_

| **Acylglycerol** | **Precursor ion (m/z)** | **Formula** | **MS^2^ product ions; formula** |
| --- | --- | --- | --- |
| PPP | 829.789 | [C_51_H_98_O_6_+Na]^+^ | *829.7;* [M+Na]^+^  *573.5;* [M-C_16_H_31_O_2_+Na]^+^  *551.5*; [M-C_16_H_31_O_2_]^+^ |
| POP | 855.755 | [C_53_H_100_O_6_+Na]^+^ | *855.7;* [M+Na]^+^  *599.5;* [M-C_16_H_31_O_2_+Na]^+^  *577.5;* [M-C_16_H_31_O_2_]^+^  *573.4;* [M-C_18_H_33_O_2_+Na]^+^  *551.5;* [M-C_18_H_33_O_2_]^+^ |
| PPS | 857.729 | [C_52_H_102_O_6_+Na]^+^ | *857.7;* [M+Na]^+^  *601.5;* [M-C_16_H_31_O_2_+Na]^+^  *579.5;* [M-C_16_H_31_O_2_]^+^  *573.5;* [M-C_18_H_35_O_2_+Na]^+^  *551.5;* [M-C_18_H_35_O_2_]^+^ |
| OSP | 883.788 | [C_55_H_104_O_6_+Na]^+^ | 883.7; [M+Na]^+^  627.5; [M-C_16_H_31_O_2_+Na]^+^  605.5; [M-C_16_H_31_O_2_]^+^  601.5; [M-C_18_H_33_O_2_+Na]^+^  599.5; [M-C_18_H_35_O_2_+Na]^+^  579.5; [M-C_18_H_33_O_2_]^+^  577.5; [M-C_18_H_35_O_2_]^+^ |
| PSS | 885.781 | [C_55_H_106_O_6_+Na]^+^ | 885.7; [M+Na]^+^  629.5; [M-C_16_H_31_O_2_+Na]^+^  607.5; [M-C_16_H_31_O_2_]^+^  601.5; [M-C_18_H_35_O_2_+Na]^+^  579.5; [M-C_18_H_35_O_2_]^+^ |
| OSS | 911.801 | [C_57_H_108_O_6_+Na]^+^ | 911.8; [M+Na]^+^  629.6; [M-C_18_H_33_O_2_+Na]^+^  627.6; [M-C_18_H_35_O_2_+Na]^+^  607.5; [M-C_18_H_33_O_2_]^+^  605.5; [M-C_18_H_35_O_2_]^+^ |
| SSS | 913.817 | [C_57_H_110_O_6_+Na]^+^ | 913.8; [M+Na]^+^  629.6; [M-C_18_H_33_O_2_+Na]^+^  607.5; [M-C_18_H_33_O_2_]^+^ |
| AOO | 937.798 | [C_59_H_110_O_6_+Na]^+^ | 937.7; [M+Na]^+^  655.7; [M-C_18_H_33_O_2_+Na]^+^  633.7; [M-C_18_H_33_O_2_]^+^  625.7; [M-C_20_H_39_O_2_+Na]^+^  603.7; [M-C_20_H_39_O_2_]^+^ |
| BOO | 965.889 | [C_61_H_114_O_6_+Na]^+^ | 965.9; [M+Na]^+^  683.9; [M-C_18_H_33_O_2_+Na]^+^  661.7; [M-C_18_H_33_O_2_]^+^  625.8; [M-C_22_H_43_O_2_+Na]^+^  603.7; [M-C_22_H_43_O_2_]^+^ |
| C_18:2,OH_PP | *869.714* | C_53_H_100_O_7_+Na]^+^ | 869.7; [M+Na]^+^  613.5; [M-C_16_H_31_O_2_+Na]^+^  573.5; [M-C_18_H_33_O_3_+Na]^+^  551.5; [M-C_18_H_33_O_3_]^+^  319.2; [C_18_H_31_O_3_+Na]^+^ |
| C_18:2,OH_OP | *895.714* | [C_55_H_100_O_7_+Na]^+^ | 895.7; [M+Na]^+^  639.5; [M-C_16_H_31_O_2_+Na]^+^  613.5; [M-C_18_H_33_O_2_+Na]^+^  599.5; [M-C_18_H_31_O_3_+Na]^+^  577.5; [M-C_18_H_31_O_3_]^+^  319.2; [C_18_H_31_O_3_+Na]^+^ |
| C_18:2,OH_C_18:2,OH_P | *909.706* | [C_55_H_98_O_8_+Na]^+^ | 909.7; [M+Na]^+^  653.5; [M-C_16_H_31_O_2_+Na]^+^  613.5; [M-C_18_H_31_O_3_+Na]^+^  319.2; [C_18_H_31_O_3_+Na]^+^ |
| C_18:2,OH_C_18:2,OH_S | *937.705* | [C_57_H_102_O_8_+Na]^+^ | 937.7; [M+Na]^+^  653.5; [M-C_18_H_35_O_2_+Na]^+^  641.5; [M-C_18_H_31_O_3_+Na]^+^  319.2; [C_18_H_31_O_3_+Na]^+^ |

**Table S.2 -** List of the 44 pastels and summary of the XRF results. Elements are in order of decreasing XRF signal intensity (elements with the highest XRF signals are reported in bold, elements with peak intensity less than 200 counts are reported in brackets); *LF= Le Franc; DS= Dr.F. Schoenfeld.

| **Name*** | **Color** | **XRF results** |
| --- | --- | --- |
| LF 01 | red/brown | **Fe**,**Ba**, Zn,As,Sr,S (Ca,Si) |
| LF 02 |  | **Zn**,Fe, Ba,Ca (S,Pb,Sr) |
| LF 03 |  | **Hg**,S,Ca |
| LF 04 |  | **Zn**,S (Ca,Fe,Ti,Al,Pb) |
| LF 05 |  | **Fe**,K, Zn (Ti,Pb,Si,Ca,Mn) |
| LF 06 |  | **Zn**,Fe,Ba (Pb/As,S,Al,Mn) |
| DS 17 |  | **Fe**,**Ba,**Ca,Sr,S,Pb,Zn (Si) |
| DS 18 |  | **Zn**,Fe,Ba (S,Ca,Pb,Sr) |
| DS 19 |  | **Zn**,Hg (Ca,Ba,Fe) |
| DS 30 |  | **Fe,** Ba,Sr,S (Ca,As,Si,Cu,Zn) |
| DS 34 |  | **P,S**,Fe,Zn,Al (Cu,Ti) |
| LF 55 |  | **Zn**,**Fe**,Ba (Sr,S,As/Pb?) |
| DS 57 |  | **Fe**,Pb,Cr,Ca,Ti,K,Si,Sr(Al, Zn) |
| DS 29 | grey/brown | **Zn**,Fe,Ba (Pb,Ca) |
| DS 33 |  | **Zn,**Fe,Ba (Pb,Ca,S?) |
| DS 37 | purple | **Zn,**Fe,S (Ba,Ca,Pb,Hg) |
| DS 38 |  | **Zn** Mn,P,Pb (Fe,Ba) |
| DS 71 |  | **S,** Fe, Zn,Ti,Ca (Si,Al) |
| LF 14 | blue | **Zn** Fe,S,Ca (Si,K,Ba/Ti,Pb,Al) |
| LF 15 |  | **Zn,**Pb,As,Cu,Fe (Ca, Cr, Ti/Ba?) |
| DS 27 |  | **Zn,**Cu,As, Fe (Pb,Ca, Ba, Si) |
| DS 28 |  | **Zn,**Fe (Ba,Ca,S,Si) |
| DS 63 |  | **Zn**,(Fe,Ba,Cu) |
| LF 07 | yellow | **Zn** (Fe,Pb,Cr,Ba) |
| LF 08 |  | **Pb**,Cr,Ca |
| LF 09 |  | **Zn**,Pb Cr(Fe, Ca,Ti/Ba?) |
| LF 10 |  | **Zn,**Pb,Cr,Ca (Fe,Ti/Ba?) |
| DS 20 |  | **Pb,**Cr,Ca (Fe,Zn,Ti/Ba?) |
| DS 21 |  | **Zn** (Pb,Cr,Fe, Ca,Ti/Ba?) |
| DS 22 |  | **Zn**,Fe,Pb,Cr,(Ca, Ba) |
| LF 11 | green | **Pb,**Fe,Cr (Ca,Zn,K) |
| LF 12 |  | **Pb,**Fe,Cr(K,Zn,Si) |
| LF 13 |  | **Pb,**Cr,Fe(Ca,Zn,Si,Ti/Ba?) |
| DS 23 |  | **Zn,Cr,**Ca (Ti/Ba?,Fe,Pb) |
| DS 24 |  | **Zn,**Pb,Cr,Fe,Ca (Ba) |
| DS 25 |  | **Zn,**Pb (Cr,Fe,Ba,Ca) |
| DS 26 |  | **Zn,Cr**(Ca,Ba,Fe,Pb) |
| DS 31 |  | **Cr,**Ca(Fe,Zn,Ti/Ba) |
| DS 32 |  | **Zn**,Cr (Ca,Fe,Ti/Ba) |
| DS 35 |  | **Zn,**Pb,Fe,Cr(Ca,Ba) |
| DS 36 |  | **Zn,**Cu,As(Fe,Ba,Pb) |
| DS 60 |  | **Zn,**Pb,Fe,Cr(Ca,Ba,Si) |
| DS 68 |  | **Zn,**Cu,As (Fe,Ba,Pb) |
| LF 16 | black | **Fe,Ba** Ca,Sr,S (P,Si,Zn,Pb?) |

**Bibliography**

[1] J. La Nasa, I. Degano, F. Modugno, M.P. Colombini, Industrial alkyd resins: characterization of pentaerythritol and phthalic acid esters using integrated mass spectrometry, Rapid Communications in Mass Spectrometry, 29 (2015) 225-237.

[2] J. La Nasa, I. Degano, F. Modugno, M.P. Colombini, Alkyd paints in art: Characterization using integrated mass spectrometry, Analytica Chimica Acta, 797 (2013) 64-80.

[3] I. Degano, J. La Nasa, E. Ghelardi, F. Modugno, M.P. Colombini, Model study of modern oil-based paint media by triacylglycerol profiling in positive and negative ionization modes, Talanta, 161 (2016) 62-70.

[4] J. La Nasa, I. Degano, F. Modugno, M.P. Colombini, Effects of acetic acid vapour on the ageing of alkyd paint layers: Multi-analytical approach for the evaluation of the degradation processes, Polymer Degradation and Stability, 105 (2014) 257-264.

[5] L. Blanco-Zubiaguirre, E. Ribechini, I. Degano, J. La Nasa, J.A. Carrero, J. Iñañez, M. Olivares, K. Castro, GC–MS and HPLC-ESI-QToF characterization of organic lipid residues from ceramic vessels used by Basque whalers from 16th to 17th centuries, Microchemical Journal, 137 (2018) 190-203.
